# Supplementary material for: Leptospira seroprevalence and associated risk factors among slaughterhouse workers in Western Bahr El Ghazal State, South Sudan
Source: PLoS Negl Trop Dis. 2024 Dec 11;18(12):e0012700. doi: 10.1371/journal.pntd.0012700 (PMC11633975; doi:10.1371/journal.pntd.0012700)
Supplement: S1 Table — (DOCX) [file pntd.0012700.s001.docx]

S 1 Table. Panel of Leptospira spp., used as live antigens in the Microscopic Agglutination Test

| **Genomospecies** | **Serogroups** | **Serovars** | **Strains** |
| --- | --- | --- | --- |
| *L. interrogans* | Icterohaemorrhagiae | Icterohaemorrhagiae | RGA |
|  | Pomona | Pomona | Pomona |
|  | Hebdomadis | Hebdomadis | Hebdomadis |
|  | Australis | Australis | Ballico |
|  | Canicola | Canicola | Strain Hond Utrecht IV |
| *L. borgpetersenii* | Sejroe | Sejroe | M84 |
|  | Ballum | Kenya | Njenga |
|  | Pyrogenes | Nigeria | Vom |
|  | Tarassovi | Tarassovi | Perepelitsin |
| *L. kirschneri* | Autumnalis | Butembo | Butembo |
|  | Grippotyphosa | Grippotyphosa | Duyster |
| *L. weilli* | Celledoni | Celledoni | Celledoni |
