# Supplementary material for: Leptospira seroprevalence and associated risk factors among slaughterhouse workers in Western Bahr El Ghazal State, South Sudan
Source: PLoS Negl Trop Dis. 2024 Dec 11;18(12):e0012700. doi: 10.1371/journal.pntd.0012700 (PMC11633975; doi:10.1371/journal.pntd.0012700)
Supplement: S2 Table — (DOCX) [file pntd.0012700.s002.docx]

S2 Table**.** Leptospiral serovar, strain, and serogroup panel, leptospiral seropositivity, determined by the Microscopic Agglutination Test (titer ≥ 100), among slaughterhouse workers (N = 250) sampled in Western Bahr El Ghazal State, South Sudan.

| ***Leptospira* Serovar** | **Strains** | **Serogroup** | | **MAT titer** | | | | | | | |  |  | **95% CI** | |
| --- | --- | --- | --- | --- | --- | --- | --- | --- | --- | --- | --- | --- | --- | --- | --- |
|  |  |  |  | **100** | **200** | **400** | **800** | **1600** | **3200** | **6400** | **12800** | **n Positive** | **Prevalence*** | **Lower** | **Upper** |
| *L. borgpetersenii* sv Tarassovi | RGA | Tarassovi | | 0 | 2 | 4 | 0 | 0 | 0 | 0 | 0 | 6 | 2.4 % | 0.4 | 4.4 |
| *L. interrogans* sv Australis*** | Pomona | Australis | | 2 | 2 | 1 | 1 | 0 | 0 | 0 | 0 | 6 | 2.4 % | 0.8 | 4.4 |
| *L. kirschneri sv* Grippotyphosa*** | Hebdomadis | Grippotyphosa | | 3 | 0 | 0 | 0 | 0 | 0 | 0 | 0 | 3 | 1.2 % | 0.0 | 2.8 |
| *L. borgpetersenii* sv Kenya | Ballico | Ballum | | 0 | 1 | 0 | 1 | 0 | 0 | 0 | 0 | 2 | 0.8 % | 0.0 | 2.0 |
| *L. interrogans* sv Hebdomadis*** | Strain Hond Utrecht IV | Hebdomadis | | 1 | 0 | 0 | 0 | 0 | 0 | 0 | 0 | 1 | 0.4 % | 0.0 | 1.2 |
| *L. kirschneri* sv Butembo | M84 | Autumnalis | | 0 | 0 | 0 | 0 | 0 | 0 | 0 | 0 | 0 | 0.0 % | - | - |
| *L. interrogans* sv Pomona | Njenga | Pomona | | 0 | 0 | 0 | 0 | 0 | 0 | 0 | 0 | 0 | 0.0 % | - | - |
| *L. borgpetersenii sv* Sejroe | Vom | Sejroe | | 0 | 0 | 0 | 0 | 0 | 0 | 0 | 0 | 0 | 0.0 % | - | - |
| *L. borgpetersenii* sv Nigeria | Perepelitsin | Pyrogenes | | 0 | 0 | 0 | 0 | 0 | 0 | 0 | 0 | 0 | 0.0 % | - | - |
| *L. interrogans* sv Canicola | Butembo | Canicola | | 0 | 0 | 0 | 0 | 0 | 0 | 0 | 0 | 0 | 0.0 % | - | - |
| *L. interrogans* sv Icterohaemorrhagiae | Duyster | Icterohaemorrhagiae | | 0 | 0 | 0 | 0 | 0 | 0 | 0 | 0 | 0 | 0.0 % | - | - |
| *L. weilli* sv Celledoni | Celledoni | Celledoni | | 0 | 0 | 0 | 0 | 0 | 0 | 0 | 0 | 0 | 0.0 % | - | - |
|  |  |  | | 6 | 5 | 5 | 2** | 0 | 0 | 0 | 0 | 18*** |  |  |  |
| *Any Leptospira Spp., serovar* | | | Any positive excluding cross reaction | | | | | | | |  | 16 | 6.4* | 3.2 | 10.2 |

* This is an apparent prevalence since the Microscopic Agglutination Test (MAT) is not 100% sensitive and specific.

**Probable recent leptospirosis MAT titer = 800

*** Out of the total of 16 positive sera, one cross – reacted simultaneously with the three serovars of three different serogroups.
