# Supplementary material for: Leptospira seroprevalence and associated risk factors among slaughterhouse workers in Western Bahr El Ghazal State, South Sudan
Source: PLoS Negl Trop Dis. 2024 Dec 11;18(12):e0012700. doi: 10.1371/journal.pntd.0012700 (PMC11633975; doi:10.1371/journal.pntd.0012700)
Supplement: Attachment — Submitted filename: Authors Response 240809..pdf [file pntd.0012700.s003.pdf]

9th August 2024

To: The Editor

PLOS Neglected Tropical Diseases

RE. AUTHORS' RESPONSES LETTER

We express our sincere gratitude to the editor for granting us the opportunity to revise our manuscript entitled "Seroprevalence and associated risk factors of *Leptospira* spp. among slaughterhouse workers in Western Bahr El Ghazal State, South Sudan".

We really appreciate the peer reviewers' hard work and insightful feedback, which have greatly improved our manuscript. We have addressed the reviewers' comments to the best of our ability and hence, we would like to resubmit the revised manuscript. Find below in a table the authors response to the reviewers' comments. Thanks.

<table>
<tr><td>S/No</td><td>Reviewer comments</td><td>Authors Response</td></tr>
<tr><td colspan="3">Reviewer # 1</td></tr>
<tr><td rowspan="2">1</td><td colspan="2">Abstract results</td></tr>
<tr><td>Is there something missing here? 'no cases of leptospirosis were considered' should it read 'no cases of leptospirosis had been considered'</td><td>Yes, we agree it should read as no cases of leptospirosis had been considered. This has also been corrected in the manuscript on page 2, line 44.</td></tr>
<tr><td rowspan="2">2</td><td colspan="2">Author Summary</td></tr>
<tr><td>Consider rephrasing to say 'presenting with fever' instead of 'presenting fever'</td><td>The point is addressed on page 4, line 80 of the manuscript.</td></tr>
<tr><td rowspan="2">3</td><td colspan="2">Introduction</td></tr>
<tr><td>Check reference formatting to make it uniform to the rest of the manuscript 'leptospirosis is in Africa estimated at 95.5 per 100,000 people (Mgode et al., 2015).'</td><td>The reference formatting is corrected on page 5, line 93 of the manuscript.</td></tr>
<tr><td rowspan="2">4</td><td colspan="2">Discussion</td></tr>
<tr><td>Check this sentence for grammatical correctness. 'handle at less' sounds off.</td><td>Leptospira spp. increases by (OR) of 2.3 times compared to persons who handle one</td></tr>
</table>

	'Leptospira spp. increases by (OR) of 2.3 times compared to persons who handle at less one animal'.	or fewer animals per day. This point was also addressed in the manuscript on page 21, lines 308 – 309.
5	**Methods/ data analysis**	
	Here there is reference to bivariable analysis being used to evaluate association between seroprevalence and various exposures. Was this meant to be univariable logistic regression where one variable is modeled against a single outcome? If not, kindly clarify the components of the bivariable logistic regression.	Yes, we are refereeing to bivariable logistic regression to mean the same as univariable logistic regression, as exactly you have explained. But for clarity we have replaced bivariable with univariable in the manuscript.
6	**Conclusion**	
	It is important to include the well-known limitation for cross-sectional studies which is the inability to determine causality/causal associations between variables and occurrence of leptospirosis. Consider recommending a longitudinal study for more robust understanding of these relationships.	To address the limitation of a cross-sectional study design, which prevents establishing a causal relationship with leptospirosis, we propose further longitudinal surveys for a more robust understanding of these associations. Another limitation of this study is that our results cannot be generalized to the wider population. Therefore, we recommend conducting seroepidemiological studies involving the general public and farmers. Additionally, the MAT diagnostic tool used in this study can only detect past or recent exposures. To mitigate this limitation, we recommend incorporating molecular diagnostic tools such as PCR for febrile hospitalized patients in future studies.

		This valuable comment is addressed in the manuscript on page 22, lines 336 - 343.
Reviewer #2		
7	There is some redundancy in a few sentences, e.g., presence of leptospiral antibody seropositivity (should be changed to leptospiral antibodies or leptospiral seropositivity).	Leptospiral antibodies or leptospiral seropositivity have been interchangeably used in few sentences in the manuscript and are highlighted in yellow with thanks.
8	In the manuscript, wherever percentages are presented, please consider providing raw numbers as well.	Raw numbers are provided in the manuscript and are highlighted in yellow.
9	Information in Table 1 and 3 can be combined.	Table 1 has been made as S1. While 1&3 combined has been made as S2, and we have maintained Table 3 with only five positive reacted serovars on page 14, lines 220 – 225.
10	Table 3 and 4 can be made 'supplementary figures'	Table 3 is made as S2, and we feel like Table 4 is necessary to be in the manuscript since it illustrates the associated risk factors.
11	Screening of a subset of animals from the slaughterhouses for renal carriage and/or seroprevalence would make a good future study.	Screening of a subset of animals from the slaughterhouses for renal carriage and/or seroprevalence was done and is being published and has been cited here in this manuscript as reference No. 16.

David Onafruo Kal
Department of Biosecurity, Ecosystems and Veterinary Public Health
Makerere University, Kampala Uganda, and Department of Clinical Studies University of Bahr El Ghazal , Wau South Sudan.
