# Supplementary material for: Leptospira seroprevalence and associated risk factors among slaughterhouse workers in Western Bahr El Ghazal State, South Sudan
Source: PLoS Negl Trop Dis. 2024 Dec 11;18(12):e0012700. doi: 10.1371/journal.pntd.0012700 (PMC11633975; doi:10.1371/journal.pntd.0012700)
Supplement: Attachment — Submitted filename: Authors response.pdf [file pntd.0012700.s004.pdf]

18th October 2024

To: The Editor

PLOS Neglected Tropical Diseases

RE. AUTHORS' RESPONSES LETTER

We express our sincere gratitude to the editor for granting us another opportunity to revise our manuscript entitled "Seroprevalence and associated risk factors of *Leptospira* spp. among slaughterhouse workers in Western Bahr El Ghazal State, South Sudan".

We really appreciate the peer reviewers' hard work and insightful feedback, which have greatly improved our manuscript. We have addressed the reviewers' comments to the best of our ability and hence, we would like to resubmit the revised manuscript. Find below in a table the author's response to the reviewers' comments. Thanks.

S/No	Reviewer comments	Authors Response
Reviewer # 1		
1	Consider moving the limitations section of the conclusion to the last part of the discussion. The conclusion needs to be as brief as possible and only containing key take away and recommendations.	The limitations section has been moved to the last part of the discussion section on page 21, lines 326 – 332.<br>The conclusion section is now brief with key takeaways and recommendations found on page 21, lines 334 – 341.
2	The are several typographical and grammatical issues that need addressing	This has been addressed throughout the manuscript with thanks.
3	L41: Check punctuation in this sentence after 'month'	This has been checked and corrected in the manuscript on page 2, line 41.
4	L48: I think the more appropriate way of phrasing this sentence is to say that 'people who wore aprons were 10.6 time more likely…..'	people who wore an apron/overall were 10.6 times (95% CI, 1.6 – 67.6) more likely to be seropositive than people who did not wear apron/overall. Page 3, lines 48 – 49.
5	L50: Consider replacing 'from' with 'by' for grammatical correctness	This has been considered and corrected in the manuscript on page 3, line 50.

6	L94: Replace ‘are’ with ‘is’ for grammatical correctness	This has been considered and corrected in the manuscript on page 5 line 94.
7	L112: It would be good to clarify why Wau Municipal was selected for this study. Was it for convenience?	Wau Municipal Council was selected for the study because it is the largest town in the greater Bahr El Ghazal region, serving as a central hub for livestock marketing. Additionally, the region's largest slaughterhouse has an average of 50 cattle slaughtered daily and is staffed with experienced veterinarians. This comment is addressed in the manuscript on page 5 lines 112 – 115.
8	L139: Replace ‘sample’ with ‘samples’ for grammatical correctness.	This was corrected in the manuscript on page 7, line 142.
9	L227: Did you mean ‘flaying’ instead of 'flying'?	Yes, we mean flaying. This has been corrected on page 14, line 226.
10	L235: Consider replacing 'from' with ‘by’ for correctness. Alternatively, this sentence can be phrased as ‘unit change in the number of carcasses handled'.	This was replaced in the manuscript on page 14, line 234.
11	L285: Consider excluding 'were' for clarity in this sentence.	This was considered excluded in the manuscript on page 19, 284.
12	L227: Was this variable considered as a proxy of wearing PPE or the act of wearing PPE itself? If it was the act itself then the extrapolation that it was a proxy should be brought up in the discussion and reported here as it were.	Yes, the variable was indeed considered a proxy for the use of PPE during risky activities at the slaughterhouse. This point is also addressed in the manuscript on page 20, lines 298–304. Thank you very much for raising this important question.
13	L270-273: Would the inability of including these workers in the study due to their busy	Yes, another limitation of this study is the busy schedule of some slaughterhouse

	schedule have introduced some bias in the study? Kindly consider commenting about this as a limitation of the study.	workers and their unwillingness to participate in the study. This was also addressed on page 21, lines 328 – 331.

David Onafruo Kal

Department of Biosecurity, Ecosystems and Veterinary Public Health

Makerere University, Kampala Uganda, and Department of Clinical Studies University of Bahr El Ghazal, Wau South Sudan.
